# A multicenter Spanish study of atropine 0.01% in childhood myopia progression

**DOI:** 10.1038/s41598-021-00923-1

**Published:** 2021-11-05

**Authors:** Inés Pérez-Flores, Beatríz Macías-Murelaga, Jesús Barrio-Barrio, Multicenter Group of Atropine Treatment for Myopia Control (GTAM), Inés Pérez Flores, Marta Valcárcel Vizcaíno, Marta García Arias, Sara Catalán López, Manuel Rodríguez Enríquez, María Iglesias Álvarez, Betty Lorente Bulnes, Matías García-Anllo Reinoso, José María Carnero, Victoria de Rojas Silva, Jesús Barrio Barrio, Beatríz Macías-Murelaga, David Rodríguez Feijoo, Javier Rodríguez Sánchez, Argentina Rosario Calvo Robles, Sonia López-Romero Moraleda, Ángela Barrajón Rodríguez, Javier Gálvez Martínez, Diana Victoria Mesa Carina, Elena Galán Risueño, Esther Rodríguez Domingo

**Affiliations:** 1 Department of Ophthalmology, Ribera Povisa Hospital, Vigo, Spain; 2 Department of Ophthalmology, Alava University Hospital, Vitoria, Spain; 3 Department of Ophthalmology, Navarra University Clinic Hospital, Navarra Institute for Health Research, IdiSNA, Pio XII, 36. Pamplona, 31008 Navarra, ES Spain; 4 Department of Ophthalmology, Vigo University Hospital, Vigo, Spain; 5 Department of Ophthalmology, Lugo University Hospital, Lugo, Spain; 6 Department of Ophthalmology, Ourense University Hospital, Ourense, Spain; 7 Lorente Clinic, Ourense, Spain; 8 Department of Ophthalmology, A Coruña University Hospital, A Coruña, Spain; 9 Victoria de Rojas Ophthalmologic Institute, A Coruña, Spain; 10 Las Claras Clinic, Salamanca, Spain; 11 Department of Ophthalmology, La Mancha Centro General Hospital, Alcázar de San Juan, Ciudad Real Spain; 12 Department of Ophthalmology, Leon University Hospital, Leon, Spain

**Keywords:** Eye diseases, Vision disorders

## Abstract

To evaluate the efficacy and safety of atropine 0.01% eye drops for myopia control in a multicentric pediatric Spanish cohort. An interventional, prospective, multicenter study was designed. Children aged between 6 and 14 years, with myopia between − 2.00 D to − 6.00 D, astigmatism < 1.50 D and documented previous annual progression greater than − 0.5 D (cycloplegic spherical equivalent, SE) were included. Once nightly atropine 0.01% eye drops in each eye were prescribed to all participants for 12 months. Age, gender, ethnicity and iris color were registered. All patients underwent the same follow-up protocol in every center: baseline visit, telephone consultation 2 weeks later and office controls at 4, 8 and 12 months. At each visit, best-corrected visual acuity, and cycloplegic autorefraction were assessed. Axial length (AL), anterior chamber depth and pupil diameter were measured on an IOL Master (Carl Zeiss Meditec, Inc, Dublin, CA). Adverse effects were registered in a specific questionnaire. Mean changes in cycloplegic SE and AL in the 12 months follow-up were analyzed. SE progression during treatment was compared with the SE progression in the year before enrollment for each patient. Correlation between SE and AL, and annual progression distribution were evaluated. Progression risk factors were analyzed by multivariate logistic regression analyses. Of the 105 recruited children, 92 completed the treatment. Mean SE and AL changes were − 0.44 ± 0.41 D and 0.27 ± 0.20 mm respectively. Mean SE progression was lower than the year before treatment (− 0.44 ± 0.41 D versus − 1.01 ± 0.38 D; p < 0.0001). An inverse correlation between SE progression and AL progression (r: − 0.42; p < 0.0001) was found. Fifty-seven patients (62%) had a SE progression less than − 0.50 D. No risk factors associated with progression could be identified in multivariate analyses. Mean pupil diameter increment at 12-months visit was 0.74 ± 1.76 mm. The adverse effects were mild and infrequent, and decreased over the time. Atropine 0.01% is effective and safe for myopia progression control in a multicentric Spanish children cohort. We believe this efficacy might be extensible to the myopic pediatric population from Western countries with similar social and demographic features. More studies about myopia progression risk factors among atropine treated patients are needed.

## Introduction

Myopia and its progression causes are still not well known, and that is why not only prevalence increment factors but also its possible management remain a source of interest.

In 2000 the global prevalence of myopia was 23% and of high myopia 3%. However, by 2050 these proportions will raise respectively to 50% (5 billion) and 10% (1 billion) of the world population^1^.

Demographic studies show higher prevalence in urban cores and highlight multifactor influence: ethnic, environmental and socio-economic. For instance, while myopia prevalence in Africa is lower than 10% and in Western countries is between 20 and 40%, in East Asia the prevalence of myopia is 85% and of high myopia 20% at the age of 18^2–4^.

The main concern for this increment, especially of high myopia, is based on the derived risks and costs of the associated complications, that could be the cause of irreversible low vision of almost a 7% rate in Europe up to 30% in Asia^4,5^. Despite high myopic patients are those with higher risk, mild and moderate myopia involves also an increased prevalence of potentially severe visual complications, especially myopia associated maculopathy^6^. Irreversible visual impairment risk increases with higher axial length (AL)^7^.

During the last decade, several systematic reviews and clinical trials suggest that effective therapeutic alternatives could be applied for slow myopia progression in clinical practice. Systematic reviews asses the multiple developed treatments and conclude that atropine is nowadays the most effective one^8^. Atropine is the most studied and used antimuscarinic agent for myopia management. It is believed that its fundamental action is produced by blocking the muscarinic receptors of the retina and of the scleral fibroblasts, acting as an ocular growth inhibition factor^8–11^. There is sufficient evidence that atropine 0.01% treatment is an effective first-line alternative for the control of myopia progression in children^12^.

The “*Atropine for the Treatment of Myopia” *(*ATOM*) clinical trials performed in Singapore, offered valuable information of the atropine effect in myopia progression. The ATOM 1 study showed that atropine 1% eyedrops were effective in controlling myopic progression over two years but with visual side effects that compromised treatment compliance ^13^. In phase 1 of the ATOM 2 study, lower atropine doses were studied (0.5%, 0.1%, 0.01%) and the 0.01% dose resulted comparatively effective with minimal side effects. After one washout year (ATOM 2: phase 2), patients treated with atropine 0.01% suffered less rebound phenomenon and better response after reintroducing the treatment at the lowest dose. At five years atropine 0.01% treated patients, were the ones with less myopic progression (ATOM 2 Phase 3)^14–16^.

Subsequently, “*Low-Concentration Atropine for Myopia Progression*” (LAMP) study, conducted in Hong-Kong (China), evaluated the efficacy and safety of low-concentration atropine eyedrops at 0.05%, 0.025% and 0.01% compared with placebo. They concluded that the effectiveness was concentration-dependent, obtaining better response using atropine 0.05% with similar effect over the first and second year of treatment. In contrast, and like what happened in ATOM studies, 0.01% atropine efficacy during the second year improved compared to the first^17,18^. Recently, another clinical trial conducted in Chinese children has confirmed the efficacy of atropine 0.01% in reducing myopia and AL progression^19^.

Most of the clinical trials and studies published to date involving atropine for myopia management have been performed in Asian population and there are reasonable doubts concerning efficacy and security comparing Asian to Caucasian children^12,20^. Nevertheless, the implementation of the Asian clinical trial protocols among the clinical practice of the Western countries ophthalmologists is increasing, being the 0.01% dose the most used one^21^. The purpose of this study was to analyze the effectiveness and safety of 0.01% atropine treatment for one year in a multicentric sample of children from Spain.

## Patients and methods

This multicenter interventional prospective study included Spanish children aged 6 to 14 years with refractive error from − 2.00 to − 6.00 D, astigmatism less than 1.50 D, and documented myopic progression of at least − 0.50 D under cycloplegic examination over the previous year.

Patients were recruited from October 2017 to April 2019 in 12 Spanish centers: POVISA Hospital, Vigo; Vigo University Hospital Complex; Lugo University Hospital; Ourense University Hospital Complex; Lorente Clinic (Ourense); A Coruña University Hospital Complex; Victoria de Rojas Ophthalmologic Institute (A Coruña); Navarra University Clinic; Alava University Hospital; Las Claras Clinic (Salamanca); Leon University Assistance Complex; and La Mancha Centro General Hospital.

In each center, consecutive myopic children that accepted to participate in the study and met inclusion criteria requirements were enrolled, and atropine 0.01% was prescribed for their myopia progression management. All included patients were treated with 0.01% atropine sulfate one nightly eyedrop in each eye for 12 months. Neither randomization nor placebo nor control group were established. The study was classified by The Spanish Agency of Medicines and Medical Devices (AEMPS) as an observational prospective post-authorization study (Classification Code: IPF-ATR-2017-01 on October 26, 2017).

The study was approved by the referral Ethics Committee corresponding to each of the participating centers: *Comité de Ética de la Investigación con medicamentos (CEIm) Autonómico de Galicia, CEIm de la Comunidad Foral de Navarra, CEIm Área de Salud de León y El Bierzo, CEIm Área de Salud de Salamanca, CEIm Gerencia de Atención Integrada de Alcázar de San Juan (Ciudad Real), CEIm de Euskadi*. Written informed consent was obtained from parents or legal tutors of all participants. All procedures were conducted according to the tenets of the Declaration of Helsinki.

The eyedrops were compounded and dispensed in AEMPS authorized pharmacies in accordance with an identical procedure. The 0.01% atropine ophthalmic solution was prepared in a sterile manner (Atropine Sulfate 1 mg, Sodium Chloride ClNa 0.9%, Glacial Acetic Acid q.s., Sodium Acetate q.s. to pH 5.0–6.0; Active Pharmaceutical Ingredient API 10 ml), and was stored in Low Density Poliethylene LDPE multi-dose bottles.

Patients with ocular or systemic diseases that could affect vision or refractive error, contraindicated use of atropine due to any reason, amblyopia or strabismus history, previous use of atropine or pirenzepine, orthokeratology lens for myopia control or any other circumstances that could impede protocol adhesion, including the refusal to stop using contact lenses during the duration of the study, were excluded.

The same study and exploration protocols were followed in each hospital. Age, gender, ethnicity, and iris color were registered in every patient. At each visit, best-corrected distance and near visual acuity was assessed according to logMAR scale, using Early Treatment Diabetic Retinopaty Study (ETDRS) charts. Ocular AL and anterior chamber (AC) depth were measured on an IOL Master (Carl Zeiss Meditec, Inc, Dublin, CA), with six readings of average. Automatized measures of pupil diameter (IOL Master, Zeiss) were made with the same ambient light conditions. Cycloplegic autorefraction (Nidek ARK-510, Nidek) was performed at least thirty minutes after the third 1% cyclopentolate eyedrop, and three to five readings of the spherical and cylinder components that had to be less than < 0.25 D apart were obtained. Spherical equivalent (SE) was calculated as spherical power plus half of the cylinder power. When necessary, cycloplegic subjective refraction was done for glasses prescription. All the patients underwent the same follow-up protocol: after the initial visit, a telephone consultation was provided two weeks later concerning to the treatment tolerance and compliance; then the patients had office controls at 4, 8 and 12 months from the baseline visit.

Compliance and treatment side effects were evaluated verbally with the parents by telephone call two weeks after baseline visit, and with both, parents and children, during the next visits. A questionnaire was designed to document local side effects (ocular discomfort, light intolerance and blurred near vision) quantified as mild, moderate or severe. Systemic adverse effects were registered as present or absent as well. If present, the type of systemic pathology and possible relation with the use of atropine, were analysed.

The main outcome of this study was myopia progression in terms of SE and AL changes over one year. The SE progression was categorized as ≤ − 0.50 D; − 0.50 < X < − 0.99 D and ≥ − 1.00 D, and patient distribution was analyzed depending on the result at twelve months. Possible risk factors associated with progression > − 0.50 D were also analyzed.

### Statistical analysis

Sample size was calculated as follows. The children population between 6 to 14-year-old in Spain corresponded to 4,380,101 individuals (*Spanish Statistical Office, National Statistics Institute, Jan 2017*). Assuming a myopia prevalence among 10-year-old children of white ethnicity of 6.7%^3^, our Spanish reference population would be of 293,467 myopic children. We set a safety level of 95% and a degree of accuracy of 5%, which gave us a calculated sample size of 384 individuals. We placed our sample horizon in 400 participants allowing a 5% attrition rate.

Correlation analysis was performed with data of both eyes that permitted a pooled analysis of those values as a unique data for each patient. A descriptive analysis of the variables was also performed. Change in the evaluated parameters (SE, AL, AC, pupil diameter, BCVA at distance and near) was considered as the difference between the baseline data and the data at the end of the follow-up period. Lilliefors test was used to prove the statistical assumption of normality whereas t Student and Wilcoxon test were used to prove paired samples.

Number of successes, defined as a progression < − 0.50 D and the annual percentage distribution were calculated. Pearson correlation was used to analyze the relation between the SE and AL progression. Myopia > − 0.50 D progression risk factors were studied using a multivariable analysis of logistic regression and the Huber-White robust standard error technique.

The software used for the analysis was: R (3.5.1. version), RStudio (1.1.463 version) and IBM SPSS Statistics 20. P value < 0.05 was considered statistically significant.

## Results

### Patients

A total of 105 patients that met inclusion criteria were recruited. During the study period, 13 subjects were lost: 4 due to treatment side effects, 1 due to orthokeratology treatment introduction and 8 that did not attend the follow-up visits. Finally, 92 patients that finished the twelve-month treatment protocol were analyzed.

Demographic data are resumed in Table 1. Mean age was 9.76 ± 1.93 years (6–14 years), 46 (50%) were female, 90 (97.8%) were Caucasian and 71 (77.2%) had brown colored iris.

**Table 1:** Demographic variables distribution.

Variable	n (%)
**Patients**	92 (100)
**Gender (female)**	46 (50)
**Ethnicity**	
Caucasian	90 (97.8)
Asian	2 (2.2)
**Iris color (pigmentation)**	
Dark	71 (77.2)
Medium	14 (15.2)
Light	6 (6.6)

### Ocular parameters

**Figure 1:**
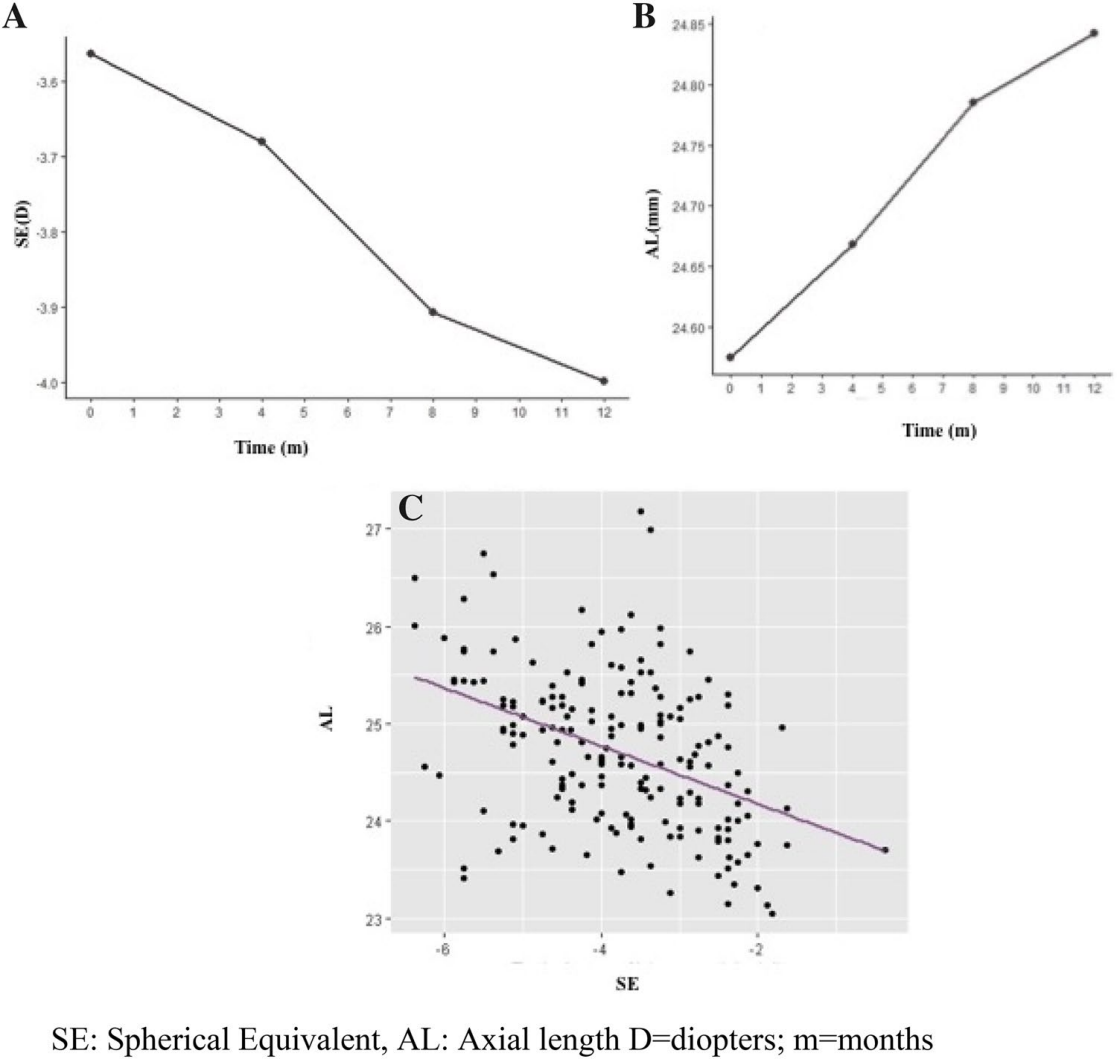
A Change in spherical equivalent across the time. B Change in axial length across the time. C Spherical equivalent and axial length progression correlation (Pearson Correlation r: − 0.42; p < 0.0001). *SE* Spherical Equivalent, *AL* Axial length *D* diopters, *m* months.

After the first year of treatment, mean increase in the SE was of − 0.44 ± 0.41 D and of 0.27 ± 0.20 mm in AL. Mean SE progression significantly decreased comparing with the progression of the previous year (− 0.44 ± 0.41 D versus − 1.01 ± 0.38 D). The AL change could not be calculated because we did not have AL data documented from the previous year. The correlation between the SE and the AL during the first year was moderate (r: − 0.42; p < 0.0001) (Fig. 1). Table 2 shows the changes in the ocular parameters at the end of the study. All parameters underwent significantly changes except for best corrected visual acuity at distance and near.

**Table 2:** Changes in ophthalmic parameters after one-year atropine 0.01% treatment.

Variable	Baseline visit	12 month visit	Mean change at 12 month	p-value*
SE (mean ± SD, D)	− 3.56 ± 1.12	− 4.00 ± 1.14	− 0.44 ± 0.41	0.0000
AL (mean ± SD, mm)	24.57 ± 0.79	24.84 ± 0.80	0.27 ± 0.20	0.0000
AC (mean ± SD, mm)	3.80 ± 0.32	3.83 ± 0.30	0.03 ± 0.25	0.0085
Pupil size (mean ± SD, mm)	5.60 ± 1.37	6.32 ± 1.11	0.74 ± 1.26	0.0000
Near VA (mean ± SD, logMAR)	0.00 ± 0.02	0.00 ± 0.01	0.00 ± 0.03	0.8539
Distance VA (mean ± SD, logMAR)	0.00 ± 0.03	− 0.01 ± 0.04	− 0.01 ± 0.05	0.3583

*t Student and Wilcoxon test paired samples.

*SE* Spherical Equivalent, *SD* standard deviation, *D* diopter, *AL* Axial length, *AC* Anterior chamber, *VA* Visual acuity, *logMAR* logarithm of minimum angle resolution.

**Figure 2:**
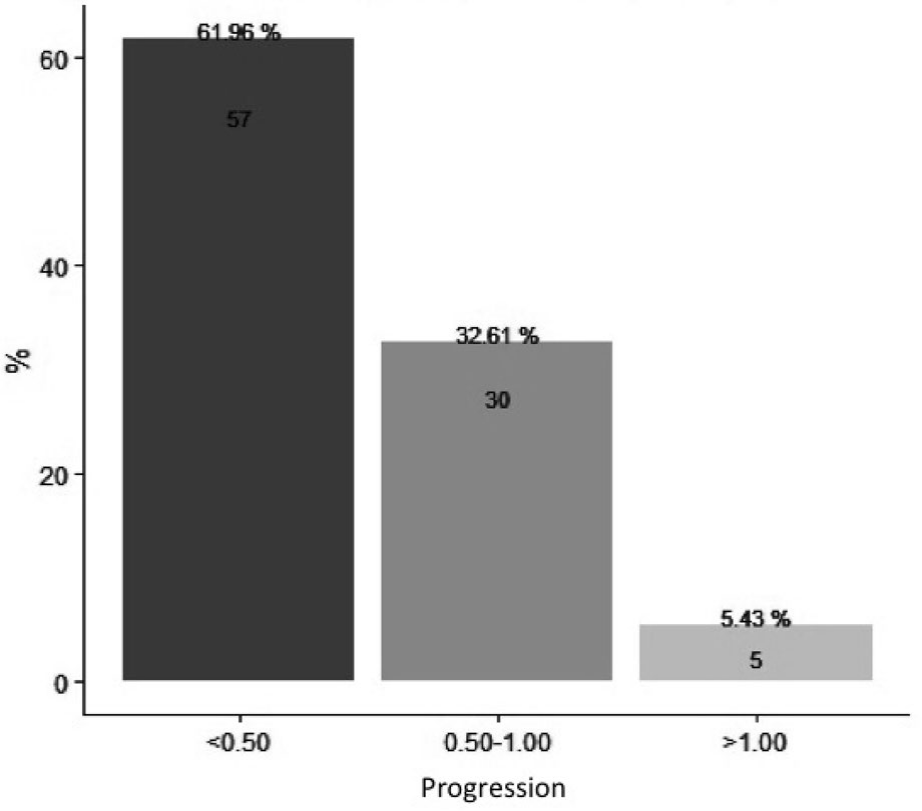
Patients distribution of the spherical equivalent progression during follow-up period.

In terms of treatment achievements, 57 (61.9%) patients underwent a progression in the SE < − 0.50 D over the first year; on the other hand, 5.4% had a progression ≥ − 1.00 D and were considered as non-responders (Fig. 2).

In the multi variable logistic regression analysis none of the analyzed baseline parameters, neither demographic (age, gender, iris color) nor ocular (progression in the previous year, SE, AL, AC, pupil size) could be significantly associated with the progression of the SE > − 0.50 D during the first year of treatment (Table 3).

**Table 3:** Progression risk factor analysis for spherical equivalent more than − 0.50 diopters.

	Odds ratio	Residual	p-value*
**Age**	0.87	0.10	0.14
**Gender**			
Female	1.08	0.10	0.14
**Iris color**			
Medium	2.12	0.33	0.81
Light	0.30	0.74	0.10
**Previous year progression**	0.54	0.40	0.12
**Spheric equivalent**	1.51	0.36	0.25
**Axial length**	1.22	0.24	0.40
**Anterior chamber**	0.84	0.43	0.67
**Pupil size**	1.09	0.10	0.38

*Multi variable logistic regression analysis and Huber-White robust standard error.

### Adverse effects

Most of the observed side effects were mild. In the survey at 2 weeks: 14 (15.2%) patients had mild ocular discomfort; there were 7 patients with light intolerance that in 6 (6.5%) was mild and in 1 (1.1%) was moderate, and 5 (5.4%) patients manifested mild near vision difficulty. At 12 months: 1 (1.1%) patient had mild ocular discomfort, 2 (2.2%) had mild light intolerance and there were 2 patients with near vision difficulty, 1 (1.1%) mild and 1 (1.1%) moderate. In 5 patients (5.4%) non-atropine associated mild systemic pathology was found at 2 weeks and in 3 (3.3%) at 4 months.

Of all the 13 excluded patients, 4 did not finish the follow up period due to adverse effects. Two of them were excluded for systemic pathology that could not be dismissed as atropine-related (one tachycardia and one vertigo) and two due to nonspecific ocular discomfort.

## Discussion

We have conducted a prospective multicenter Spanish study to evaluate the effect of atropine 0.01% in myopia progression in children quantifying the refractive error as well as the axial length. A standardized protocol was designed not only for examination and data collection, but also for the identification of possible treatment side effects via telephone survey and office visits.

This is a real-world evidence study inspired in Asian clinical trials concerning atropine use for myopia progression and that has let us transfer its efficacy results to the daily clinical practice in Spain. We had 12 participating centers covering a wide extension of our country and we achieved a good follow-up with few dropout cases. Our approach was previously developed in a study that analyzed the effectiveness and security of atropine 0.5% in Europeans, in which a very good response was observed but with a high rate of adverse effects^22^.

We found that atropine 0.01% is an effective treatment to slow down myopia progression in Spanish children. In our study, the relative reduction of refractive error progression after 1-year treatment was of 56% compared to the previous year. Such reduction was superior to the Asian clinical trials^14,17,19,23^, and similar to other non-Asian studies^24–27^ (Table 4). Several hypotheses can be suggested to explain this difference: the sample studies characteristics, the possible higher sensitivity to cycloplegic agents in less pigmented Caucasian iris and the socio-cultural and environmental differences between patients of different countries. European children spend more time outdoors, which might have influence on progression reduction comparing with Asian children^19,28^. As demonstrated during COVID confinement, children who were stable under 0.01% atropine had decreased effectiveness because of extended time indoors^29^. These findings may support the different response to atropine among patients from a different socio-cultural background. Nevertheless, the relative reduction data should always be interpreted considering the particular characteristics of each study. We should be cautious, and do not generalize the results to all the pediatric myopic population and in the long-term^30^.

**Table 4:** One year atropine 0.01% treatment clinical trials and studies results overview.

Ethnicity	Country	Design	Recruited children (n)	Included children per group [n, (group)]*	Age (mean ± SD, years)	SE at baseline (mean ± SD, D)	AL at baseline (mean ± SD, mm)	SE change in the previous year (mean ± SD, D)	After 1 year treatment			
									SE change (mean ± SD, D)	AL change (mean ± SD, mm)	SE relative reduction (%)	Progression < − 0.5D (% patients)
**Asian studies**												
Chia 2012 ATOM 2 (Phase 1)	Singapore	Clinical Trial	400	75 (A)	9.5 ± 1.5	− 4.5 ± 1.5	25.2 ± 1.0		− 0.43 ± 0.52	0.24 ± 0.19		50
				0 (C)								
Yam 2019 LAMP (Phase 1)	Hong Kong (China)	Clinical Trial	438	97 (A)	8.23 ± 1.83	− 3.77 ± 1.85	24.70 ± 0.99	− 0.81 ± 0.32	− 0.59 ± 0.61	0.36 ± 0.29	27.2	43.8
				93 (C)	8.42 ± 1.72	− 3.85 ± 1.95	24.82 ± 0.97	− 0.88 ± 0.36	− 0.81 ± 0.53	0.41 ± 0.22	27.2	
Wei, 2020	China	Clinical Trial	220	76 (A)	9.44 ± 1.80	− 2.52 ± 1.33	24.50 ± 0.76		− 0.49 ± 0.42	0.32 ± 0.19	34.2	48.7
				83 (C)	9.84 ± 1.53	− 2.64 ± 1.46	24.69 ± 0.97		− 0.76 ± 0.50	0.41 ± 0.19		
Fu, 2020	China	Clinical Trial	400	142 (A)	9.3 ± 1.9	− 2.70 ± 1.64	24.58 ± 0.74		− 0.47 ± 0.45D	0.37 ± 0.22	32.8	45.1
				120 (C)	9.5 ± 1.4	− 2.68 ± 1.42	24.55 ± 0.71		− 0.70 ± 0.60D	0.46 ± 0.35		
**Non Asian studies**												
Larkin 2019	USA	Retrospective Multicenter	198	100 (A)	9.3 ± 2.10	− 3.1 ± 1.9			− 0.2 ± 0.8			
				98 (C)	9.2 ± 2.11	− 2.8 ± 1.6			− 0.6 ± 0.4		66.6	
Sacchi 2019	Italy	Retrospective	102	52 (A)	9.7 ± 2.3	− 3 ± 2.23		− 1.20 ± 0.64	− 0.54 ± 0.61		55	79
				50 (C)	12.1 ± 2.9	− 2.63 ± 2.68		− 0.80 ± 0.64	− 1.09 ± 0.64		50.5	
Clark 2015	USA	Retrospective	60	32 (A)	10.2 ± 2.2	− 2.0 ± 1.6			− 0.1 ± 0.6		83.3	
				28 (C)	10.2 ± 2.2	− 2.0 ± 1.5			− 0.6 ± 0.4			
Joachimsen 2019	Germany	Retrospective	56	56 (A)	11	− 3.85 ± 1.88		− 1.05 ± 0.37	− 0.40 ± 0.49		65	
				0 (C)								
Pérez 2021 GTAM	Spain	Prospective Multicenter	105	92 (A)	9.76 ± 1.93	− 3.56 ± 1.12	24.57 ± 0.79	− 1.01 ± 0.38	− 0.44 ± 0.41	0.27 ± 0.20	56.43	62
				0 (C)								

*SE* Spherical Equivalent, *SD* standard deviation, *D* diopter, *AL* Axial length, *A Atropine group, *C* Control group.

Concerning the annual progression distribution, 62% of the patients presented a progression < − 0.50 D. This percentage was lower than the 79% obtained in Sacchi et al. study^26^, but as well as in their study the efficacy was superior to the Asian clinical trials (Table 4). Although another study was conducted in Spain with atropine 0.01% in myopic children, neither data of progression evolution nor distribution rate over time were provided^31^. Besides, this study had a high dropout rate, and authors conducted a “dynamic randomization”, which could have introduced a bias in the results. Lastly, different cycloplegic protocol and refractive inclusion criteria prevents comparison with other studies^32^. On the other hand, 5.4% of the patients in our study were considered as non-responders, being clearly inferior to the 25% observed in the Asian ATOM 2 and LAMP studies^13,16^.

Other studies conducted in non-Asian population have not assessed the AL progression during treatment and therefore we can only compare our results with Asian clinical trials. Although the initial AL of our patients was similar to other Asian series, the AL increase after 1-year treatment in our population was lower than several Asian trials (Table 4). In any case, we did not have information about the AL progression of the previous year to the enrollment, so we could not compare this parameter before and after treatment. Latest studies suggest that myopia control research should have as main variable the AL progression because it seems that atropine does not have any clinical effect on cornea or lens power^33^. AL can be measured more precisely than SE under cycloplegia, it is not influenced by possible refractive changes induced by atropine or other treatments as orthokeratology, and myopic complications are usually associated to AL increase^34^.

After one year of atropine 0.01% treatment, we did not find any risk factor associated to the refractive error progression, although parental myopia or outdoors time data were not assessed. Some studies have described risk factors for myopia appearance and progression^35,36^, but conclusions of progression risk factors in atropine treated children remain unclear. Youngest children, with higher initial myopia and with both myopic parents, might have greater propensity to progression despite atropine 1% treatment^37^. Similarly, in a Chinese clinical trial with atropine 0.01% the greatest progression was found among the youngest children, and the authors suggested that a higher concentration of atropine should be considered in this group^19^. This finding seems to be corroborated by LAMP study, which shows how youngest children require higher concentration to reach the same myopic progression reduction than eldest children with lower concentration^33^. Furthermore, it has been discovered that the influence of the outbreak age in myopia progression differs across populations^38^ and that annual progression as an isolated factor cannot be used to predict long-term progression^39^.

With respect to the atropine 0.01% side effects, the questionnaire that we developed allowed us to quantify their severity. We agree with other authors that adverse effects were mostly mild and transitory, not preventing from treatment compliance^14,17,27,37^. None of the patients needed photochromatic protection or optical correction for near vision. A mean increase of 0.74 mm in pupil diameter was observed, in concordance with Asian and non-Asian studies that described a range between 0.5 to 1 mm^23,40^. At the beginning of the treatment, 15% of patients presented mild ocular discomfort, 6.5% mild light intolerance, and 5.4% mild near vision difficulties; these side effects were reduced to 1–2% at 12 months. Atropine 0.01% preserves high affinity to muscarinic receptors but with low impact in pupil size and accommodation (M3 receptors), compared to the influence it might have in myopia progression (M1 and M4 receptors)^40^.

There are several limitations in our study: (1) The number of cases enrolled is moderate and far from the ideal calculated sample size. Since the prevalence of myopia is lower in the Western nations than in Asia, this is a common inherent difficulty in Western studies. At the time of our final recruitment period, the prevalence of myopia among 5 to 7-year-old Spanish children was published to be around 20%^41^. Besides, patient reluctance to be involved in clinical studies, administrative difficulties with local Health Authorities that prevented participation of several invited centers, and no funding supporting of the study were the main reasons why it took a long time to recruit a medium size sample. (2) The lack of control group is another limitation of our study. We agree with other authors that consider already proven atropine efficacy in myopia control and believe that could be unethical to design studies involving a control group^18^. Scientific evidence also makes complex from an ethical point of view, the approval of a study in pediatric population with a placebo group by our country Health Authorities. (3) Retrospective comparison of SE progression could be limited by the problem of accurate refraction and documentation of data in the past. That is why only known patients being followed-up in the same institution and with previous cycloplegic refraction were included. On the other hand, we do not believe that age-related progression reduction might be a factor that could have modified the significance of the result during the treatment year^39^. (4) Unlike financially supported clinical trials, in our study the drug was compounded in multiple local sites and therefore it could had been variations in the concentrations and quality. Although no random sampling of the drug from different sites was tested for concentration and stability, only authorized pharmacies by the Spanish Agency of Medicines compounded the drug following a strict identical written protocol. (5) Neither parental myopia nor outdoor exposure were assessed and therefore our risk factor analysis is limited.

At present, this multicenter study is in its second year of treatment and when finished, we could be able to determine if our results match with those of ATOM and LAMP studies, concerning the better response to atropine 0.01% over the second year. In the future, axial length growth graphics by ethnicity, age, and gender^42,43^, genetic risk scores^44^, objective assessment of time spent outdoors and in near vision tasks^45^, and responsiveness to different atropine concentration, might help us in the decision of when start the treatment and how evaluate its efficacy.

## Conclusions

Atropine 0.01% is effective and safe for myopia progression control in a multicentric Spanish children cohort, and we believe that this conclusion might be extensible to the myopic pediatric population of Western countries with socio-cultural and environmental similar characteristics. We consider that further studies testing different atropine concentrations and long-term evolution might be necessary to analyze progression risk factors and provide more information to customize treatment in our patients.
